# Development and application of a quadruplex TaqMan fluorescence quantitative PCR typing method for *Streptococcus suis* generalis, type 2, type 7 and type 9

**DOI:** 10.3389/fcimb.2024.1475878

**Published:** 2024-11-18

**Authors:** Haojie Wang, Jianxing Chen, Yue Sun, Tongqing An, Yue Wang, Hongyan Chen, Changqing Yu, Changyou Xia, He Zhang

**Affiliations:** ^1^ State Key Laboratory for Animal Disease Control and Prevention, Harbin Veterinary Research Institute, Chinese Academy of Agricultural Sciences, Harbin, China; ^2^ College of Life Sciences and Technology, Mudanjiang Normal University, Mudanjiang, China; ^3^ School of Advanced Agricultural Sciences, Yibin Vocational and Technical College, Yibin, China

**Keywords:** *Streptococcus suis*, type 2, type 7, type 9, quadruplex TaqMan fluorescence quantitative PCR, typing

## Abstract

**Introduction:**

*Streptococcus suis* (SS) is one of the most important pathogens causing major economic losses in the global pig farming industry and is a serious threat to public health safety. It has multiple serotypes, with poor cross-protection between serotypes, and effective typing methods are lacking.

**Methods:**

In this study, a quadruplex TaqMan fluorescence quantitative PCR assay that can differentiate between *Streptococcus suis* types 2, 7 and 9 was developed using the *gdh* gene, a generic gene for *Streptococcus suis*, and *cps2J*, *cps7H* and *cps9J*, genes encoding podocarp-associated genes for types 2, 7 and 9, respectively, as targets.

**Results:**

The method is specific enough to accurately type *Streptococcus suis* pigmentosus without detecting non-target pathogens (*Escherichia coli*, *Pasteurella multocida*, *Staphylococcus aureus*, *Streptococcus agalactiae, Streptococcus pneumoniae* and et al). The sensitivity was high, with a minimum lower detection line of 10 copies for P-SS and P-SS9, and 100 copies for P-SS2 and P-SS7. The standard curves generated showed good linearity with R^2^ of 0.999, 0.999, 0.997 and 0.998 respectively. The repeatability was good, with coefficients of variation between batch to batch and batch to batch tests ranging from 0.21% to 1.10%. Testing of 156 samples yielded 68 positive and 88 negative samples, of which the positive rate of SS was 5.77% (9/156), SS2 was 20.51% (32/156), SS7 was 8.33% (13/156) and SS9 was 9.6% (15/156), which was in line with the existing fluorescent quantitative PCR assay of 93.75%~100%, which was higher than the detection rate of conventional PCR.

**Discussion:**

The quadruplex TaqMan fluorescence quantitative PCR method of *Streptococcus suis* generic, type 2, 7 and 9 established in this study can accurately differentiate the three serotypes of *Streptococcus suis* that currently have high prevalence and pathogenicity, which is of great importance for accurate clinical prevention and treatment, epidemiological investigation and vaccine development.

## 1 Introduction

*Streptococcus suis* (*S. suis*, SS) is a multi-animal commensal pathogen that colonises the upper respiratory tract of pigs, particularly the tonsils and nasal passages, and some serotypes of SS are pathogenic, mainly through wound infection (Hatrongjit et al., 2024). They can cause a wide range of diseases in pigs, including acute septicaemia, meningitis, arthritis, endocarditis and pneumonia, posing a serious threat to the development of pig farming and public health (Jiang et al., 2024). SS has been subdivided into 35 serotypes, including types 1 to 34 and 1/2, according to its capsule antigen (CPS) (Gao et al., 2024). *Streptococcus suis* type 1 (SS1), *Streptococcus suis* type 2 (SS2), *Streptococcus suis* type 7 (SS7) and *Streptococcus suis* type 9 (SS9) are the causative agents in pigs, but SS2, SS7 and SS9 are the most pathogenic (Dong et al., 2023; Hatrongjit et al., 2023; Cao et al., 2024; de et al., 2024). Among them, SS2 is the most serious threat, classified as a zoonotic infectious disease, and is the predominant causative agent both in the population and in pig herds, with the highest pathogenicity and prevalence, and is the primary target of detection for outbreak surveillance and pathogen identification, e.g (Liu et al., 2024; Yin et al., 2024). 436 cases of SS infection were detected in Thailand from January to September 2023, of which 9 died (Tungwongjulaniam et al., 2024). In addition, with the development of intensive farming, the prevalence of SS infections in china cannot be ignored, but due to the numerous serotypes of SS, the complexity of virulence factors and the interaction and linkage of virulence factors and pathogenic mechanism are still not clear, and there is a lack of effective means used to prevent and control the disease (Scherrer et al., 2024). Therefore, the establishment of rapid and effective diagnostic methods and the accumulation of epidemiological data are of great importance for the development of vaccines and the prevention and control of the disease.

Currently, bacterial isolation and culture with remains the gold standard for the diagnosis of SS (Okuhama-Yoshida et al., 2023; Uruén et al., 2023), but the method is time-consuming, with low sensitivity and high operational requirements, which is not conducive to promotion and outbreak monitoring, and the serological method cannot differentiate between podless and self-coagulating strains (Thu et al., 2021; Dong et al., 2023). With the development of molecular biology, single or multiplex PCR (Kerdsin et al., 2014), MLST (Xia et al., 2024), single fluorescent quantitative PCR (Wang et al., 2024) and other methods have been established according to the universal gene of SS or different serotype-specific podocardial antigenic genes, which have realised the identification and typing of SS at the gene level, compensated for the inadequacy of serological typing and improved the accuracy and sensitivity of detection (Wang et al., 2024). However, these methods do not allow multiple tests to be performed simultaneously, which increases the workload and cost of epidemic surveillance. In order to interrupt the spread of epidemics in a timely manner, rapid, sensitive and accurate early detection is essential for the implementation of stringent hygiene and biosecurity measures. Therefore, this study has established a rapid, sensitive and specific quadruplex TaqMan fluorescence quantitative PCR method for the simultaneous detection of SS2, SS7, SS9 and other serotypes, which are currently the most harmful and widespread.

## 2 Materials and methods

### 2.1 Strains and clinical samples

*Streptococcus suis* type 1 (SS1), *Streptococcus suis* type 2 (SS2), *Streptococcus suis* type 7 (SS7), *Streptococcus suis* type 9 (SS9), *Streptococcus suis* type 14 (SS14), *Escherichia coli* (*E. coli*), *Pasteurella multocida* (*P. multocida*), *Staphylococcus aureus* (*S. aureus*), *Streptococcus agalactiae* (*S. agalactiae*)*, Streptococcus pneumoniae* (*S. pneumoniae*), *Actinobacillus pleuropneumoniae* (*A. pleuropneumoniae*), *Mycoplasma hyopneumoniae* (*M. hyopneumoniae*), *Enterococcus faecalis* (*E. faecalis*), *Streptococcus pyogenes* (*S. pyogenes*), *Glaesserella parasuis* (*G. parasuis*), Swine Influenza Virus (SIV), Porcine Pseudorabies Virus (PRV), Porcine Circovirus Type 2 (PCV2), Porcine Reproductive and Respiratory Syndrome Virus (PRRSV) are maintained in this laboratory. 156 pig swabs and pig tissue samples (joint fluid, tonsils, lungs) were collected from March 2023-June 2024 from selected pig farms in Heilongjiang Province.

### 2.2 Design of primers and probes

Based on the genes *gdh* (GenBank ID: AY853916.1), *cps2J*(GenBank ID: AM946016.1), *cps7H* (GenBank ID: BR001004), *cps9J* (GenBank ID: KC537370), we designed specific primers and probes using Primer Express 3.0.1 to select the conserved regions (**Table 1**). The primers were synthesised by Invitrogen (Shanghai). The 5′end of the probe was labelled with FAM, NED, ROX and Cy5 fluorescence reporter groups, and the 3′end was labelled with the corresponding MGB fluorescence quenching group.

**Table 1:** Fluorescence quantitative PCR primers and probes.

Pathogens	Gene	Sequence(5'-3')	Prohuct size(bp)
SS	*gdh*	F: GGTGTCGGTGGTCGTGAGA R: TGGCGGAGGCGTTTGT Probe:FAM- CGGTTACATGTACGGTCAA-MGB	56
SS2	*cps2J*	F: CGCAGAGCAAGATGGTAGAATAAA R: ACCGTAATTCCTTGCGTTTGA Probe:NED-CCGGTTACCAAATGG-MGB	73
SS7	*cps7H*	F: GGGCAGCTCTAACACGAAATAAG R: CTGAATCCAAGAACGCAATCC Probe:ROX- CACTAAGAAAAGCTAGAGGTAG-MGB	69
SS9	*cps9J*	F: GTGTTAAACGTTTGTTCGAGAATGA R: CGCTCTAAATACACTGTCAAAGAATTG Probe :Cy5- CGAAGCTCAGGTGGGAT-MGB	52

### 2.3 Optimisation of reaction conditions

In the 25 μL reaction system, 0.2-1.0 μL each of the upstream and downstream primers (10 μmol/L) and 0.2-1.0 μL of the probe (10 μmol/L) were added, and the annealing temperatures used were 50, 52, 54, 56, 58, 60 and 62 °C, and the number of cycles was 35, 40 and 45, respectively. The lowest cycle threshold (Ct value) and the highest ΔRn were used as indicators to obtain the optimal reaction system and procedure for quadruplex TaqMan fluorescence quantitative PCR.

### 2.4 Standard curve establishment and sensitivity test

The primers in **Table 2** were used to amplify the target genes *gdh*, *cps2J*, *cps7H* and *cps9J*, respectively, and their products were recovered and purified and ligated into the pMD-18T vector and the recombinant plasmids of SS, SS2, SS7 and SS9 were transhumanised into the *DH5α* receptor cells and the culture was expanded and extracted with the kits after correct identification of bacteriophage PCR and sequencing. Recombinant plasmids (recombinant plasmid standards were designated P-SS, P-SS2, P-SS7 and P-SS9). The concentration of the plasmid was determined using a UV spectrophotometer, and the plasmid was diluted to a final concentration in the range of 4×10^0^~4×10^10^ copies/uL and mixed in equal volumes so that the final concentrations were all in the range of 1×10^0^ copies/μl~1×10^10^ copies/μl (Each gradient is repeated three times), which was then detected using the optimised quadruplex TaqMan fluorescence quantitative PCR assay.

**Table 2:** Four sets of primers used to prepare plasmid standards.

Pathogens	Gene	Primers	Sequences (5′ end to 3′ end)	length	Accession no.
SS	*gdh*	F	CCTTATAAAGGCGGTCTTCGCTT	506 bp	AY853916.1
		R	ACTTTTGCACCAAGTTCAGTCGC		
SS2	*cps2J*	F	ATGGAAAAAGTCAGCATTATTGTACC	450 bp	MH444513.1
		R	ATTTTCATTTCCTAAGTCTCGCAC		
SS7	*cps7h*	F	GTGGAAAGAGATATGGTGGAAAGAG	480 bp	BR001004
		R	AGTCAAACACCCTGGATAGCCGT		
SS9	*cps9J*	F	TAGAGTGGTATTTTTTCGATTA	379 bp	KC537370
		R	TTAATCTAATAATAAATTATTTTC		

### 2.5 Test for specificity

SS1, SS2, SS7, SS9, SS14, *E. coli*, *P. multocida*, *S. aureus*, *S. agalactiae, S. pneumoniae*, *A. pleuropneumoniae*, *M. hyopneumoniae*, *E. faecalis*, *S. pyogenes*, *G. parasuis*, SIV, PRV, PCV2, PRRSV DNA/RNA as templates, recombinant plasmid standards P-SS, P-SS2, P-SS7 and P-SS9 were used as positive controls and sterile water as a blank control using an established quadruplex TaqMan fluorescence quantitative PCR.

### 2.6 Repeatability testing

The recombinant plasmids of SS, SS2, SS7 and SS9 with concentrations of 10^7^ copies/µL, 10^5^ copies/µL and 10^3^ copies/µL were used as templates for the inter- and intra-batch experiments and the standard deviation of the Ct value and the coefficient of variation were calculated.

### 2.7 Clinical sample testing

From March 2023 to June 2024, 156 pig swabs and tissue samples (joint fluid, tonsils, lungs) were collected from pigs in some pig farms in Heilongjiang Province who exhibited symptoms such as elevated body temperature, persistent fever, depression, decreased appetite, and difficulty breathing. Nucleic acids were extracted from the above samples using the corresponding commercially available kits and then detected using the established quadruplex TaqMan fluorescence quantitative PCR assay, while the extracted nucleic acids were detected using the reported PCR assay to compare the compliance rates (Wongsawan et al., 2015; Xin et al., 2023).

## 3 Results and analyses

### 3.1 Results of optimisation of reaction conditions

After optimising the primers, probes, annealing temperature and cycle number of quadruplex TaqMan fluorescence quantitative PCR, it was found that the optimal annealing temperature was 60°C, the final concentrations of the primers were 0.08μmol/L, 0.14μmol/L, 0. 1μmol/L, and 0.12μmol/L for SS, SS2, SS7, and SS9, respectively, and the final concentrations of the probe were 0.08μmol/L, 0.08μmol/L, 0.12μmol/L, and 0.12μmol/L, respectively, and the optimal number of cycles was 40, which resulted in the lowest Ct value and the highest fluorescence signal. Final reaction system for quantitative PCR with fourfold fluorescence (**Table 3**) and reaction conditions (**Table 4**).

**Table 3:** Quadruplex TaqMan fluorescence quantitative PCR reaction system.

Reagent	Volume (μl)
2 × Animal Detection U + Probe qPCR Super PreMix	12.5
SS-F(10 μmol/L)	0.2
SS-R(10 μmol/L)	0.2
SS-Probe(10 μmol/L)	0.2
SS2-F(10 μmol/L)	0.35
SS2-R(10 μmol/L)	0.35
SS2- Probe(10 μmol/L)	0.2
SS7-F(10 μmol/L)	0.25
SS7-R(10 μmol/L)	0.25
SS7-Probe(10 μmol/L)	0.3
SS9-F(10 μmol/L)	0.3
SS9- R(10 μmol/L)	0.3
SS9- Probe(10 μmol/L)	0.3
50 × ROX Reference Dye 2	0.5
Template DNA	2
ddH_2_O	6.8
Total	25

**Table 4:** Quadruplex fluorescence quantitative PCR reaction procedure.

Step	Time	
contamination digestion 37°C	2min	
Premutability 95°C	30s	
Denaturation 95°C	$$\begin{array}{c} 10\text{s}\\ 30\text{s} \end{array}\}40\times$$
Annealing and collection of fluorescent signals 59°C	

### 3.2 Standard curves and minimum detection limits

**Figure 1:**
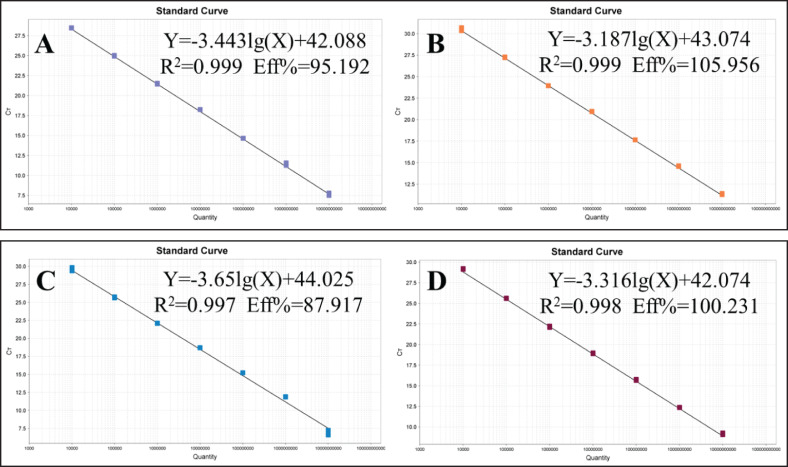
Standard curve of quadruplex TaqMan fluorescence quantitative PCR. **(A)** *Streptococcus suis generalis*; **(B)** *Streptococcus suis* type 2; **(C)** *Streptococcus suis* type 7; **(D)** *Streptococcus suis* type 9.

**Figure 2:**
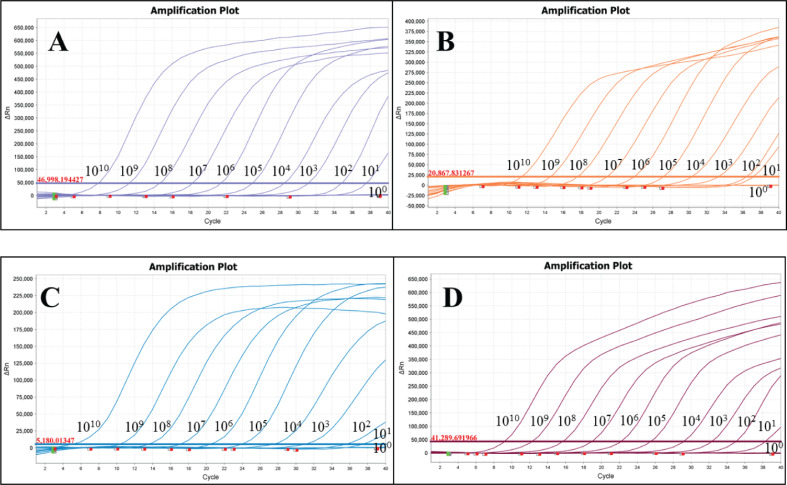
Sensitivity test of quadruplex TaqMan fluorescence quantitative PCR. **(A)** *Streptococcus suis generalis*; **(B)** *Streptococcus suis* type 2; **(C)** *Streptococcus suis* type 7; **(D)** *Streptococcus suis* type 9.

P-SS, P-SS2, P-SS7 and P-SS9 recombinant plasmid standards 1×10^10^copies/μl- 1×10^4^copies/μl were selected to plot standard curves. As shown in **Figure 1**, four standard curves were plotted and the amplification efficiencies were 95.192%, 105.956%, 87.917%, 100.231% with R^2^ of 0.999, 0.999, 0.997, 0.998, respectively. The established quadruplex TaqMan fluorescence quantitative PCR assay effectively detected a minimum concentration of 10 copies for the P-SS and P-SS9 recombinant plasmid standards, while the minimum concentration for the P-SS2 and P-SS7 recombinant plasmid standards was 100 copies (**Figure 2**). SS, SS2, SS7, and SS9 positive controls (FAM, NED, ROX, and Cy5) all had typical S-shaped amplification curves, and negative controls (FAM, NED, ROX, and Cy5) all had no amplification curves and a Ct value ≥40 or no value. The test is valid if this condition is met. If the Ct value of the test sample is <35 and a typical amplification curve appears, it is judged as positive; when 35≤Ct value <40, it is judged as suspicious and doubled for re-testing; when the Ct value is ≥40 or no value and no typical amplification curve, it is judged as negative.

### 3.3 Specificity verification results

**Figure 3:**
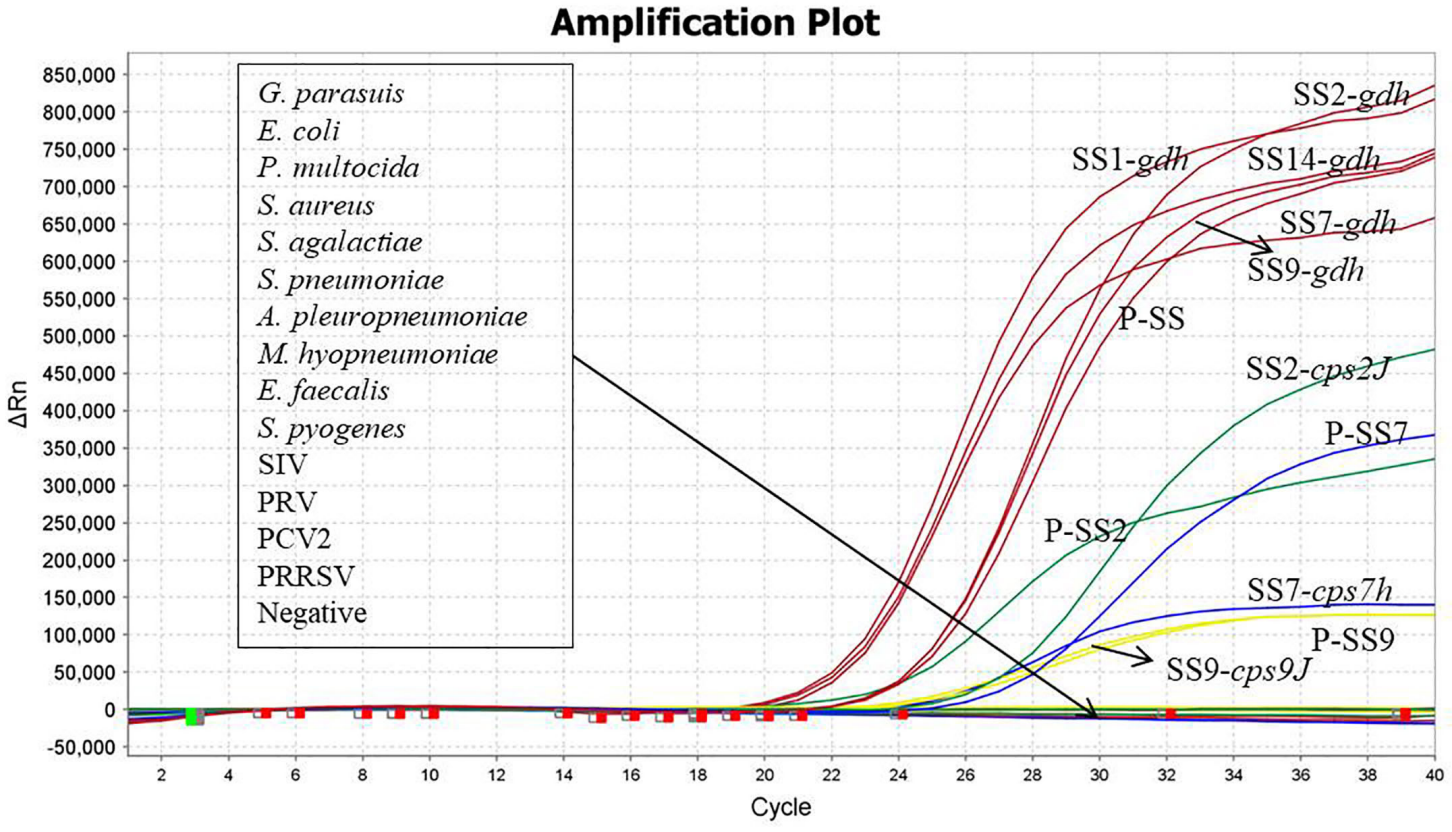
Specificity verification.

SS1, SS2, SS7, SS9, SS14, *E. coli*, *P. multocida*, *S. aureus*, *S. agalactiae, S. pneumoniae*, *A. pleuropneumoniae*, *M. hyopneumoniae*, *E. faecalis*, *S. pyogenes*, *G. parasuis*, SIV, PRV, PCV2, PRRSV were used as templates for the detection of DNA/RNA, and recombinant plasmid standards P-SS, P-SS2, P-SS7 and P-SS9 were used as positive controls. As shown in **Figure 3**, the quadruplex TaqMan fluorescence quantitative PCR assay showed typical amplification curves for SS1, SS2, SS7, SS9, SS14, and the positive control, and there were no amplification curves and Ct values for the nucleic acids of the sterile water and the non-target pathogens, indicating that the method had high specificity. The evaluation results are described in **Table 5**.

**Table 5:** Results and description of quadruplex TaqMan fluorescence quantitative PCR assay.

Pathogens	FAM	NED	ROX	Cy5
*Streptococcus suis* serotype 1	**+**	–	–	–
*Streptococcus suis* serotype 2	**+**	**+**	–	–
*Streptococcus suis* serotype 7	**+**	–	**+**	–
*Streptococcus suis* serotype 9	**+**	–	–	**+**
*Streptococcus suis* serotype 14	**+**	–	–	–
*Glaesserella parasuis*	–	–	–	–
*Escherichia coli*	–	–	–	–
*Pasteurella multocida*	–	–	–	–
*Staphylococcus aureus*	–	–	–	–
*Streptococcus agalactiae*	–	–	–	–
*Streptococcus pneumoniae*	–	–	–	–
SIV	–	–	–	–
PRV	–	–	–	–
PCV2	–	–	–	–
PRRSV	–	–	–	–
*Actinobacillus pleuropneumoniae*	–	–	–	–
*Enterococcus faecalis*	–	–	–	–
*Streptococcus pyogenes*	–	–	–	–
*Mycoplasma hyopneumoniae*	–	–	–	–

### 3.4 Repeatability test results

Recombinant plasmids of SS, SS2, SS7 and SS9 were selected at a high concentration of 10^7^, 10^5^ copies/µL and a low concentration of 10^3^ copies/µL for quadruplex TaqMan fluorescence quantitative PCR amplification, which was statistically analysed by observing the changes in the thresholds of the amplification curves and for the Ct values. The results showed that the coefficients of variation for the Ct values of SS, SS2, SS7 and SS9 were 0.21% ~ 1.10% (**Table 6**), indicating good reproducibility of this experiment.

**Table 6:** Reproducibility of the quadruplex TaqMan fluorescence quantitative PCR method.

Standard plasmid	Concentration of template (copies/μL)	Intra-coefficient of variation		Inter-coefficient of variation	
		X ± SD	CV (%)	X ± SD	CV (%)
P-SS	10^7^	17.987 ± 0.046	0.26	17.302 ± 0.036	0.21
	10^5^	24.837 ± 0.191	0.77	24.510 ± 0.086	0.35
	10^3^	31.612 ± 0.131	0.41	31.340 ± 0.122	0.39
P-SS2	10^7^	20.760 ± 0.075	0.36	20.251 ± 0.101	0.50
	10^5^	27.138 ± 0.113	0.42	27.450 ± 0.078	0.28
	10^3^	33.524 ± 0.216	0.64	34.010 ± 0.190	0.56
P-SS7	10^7^	18.420 ± 0.203	1.10	18.450 ± 0.113	0.61
	10^5^	25.750 ± 0.189	0.73	25.621 ± 0.099	0.39
	10^3^	33.075 ± 0.253	0.83	33.520 ± 0.168	0.50
P-SS9	10^7^	18.850 ± 0.092	0.50	18.862 ± 0.043	0.23
	10^5^	25.448 ± 0.170	0.67	25.984 ± 0.193	0.74
	10^3^	32.126 ± 0.133	0.41	31.990 ± 0.224	0.70

### 3.5 Clinical sample test results

A total of 156 clinical samples collected were tested simultaneously using the quadruplex TaqMan fluorescence quantitative PCR assay established in this study and the reported assays. The results showed 68 positive and 88 negative samples (**Table 7**). The SS positivity rate was 5.77% (9/156), SS2 positivity rate was 20.51% (32/156), SS7 positivity rate was 8.33% (13/156) and SS9 positivity rate was 9.6% (15/156). No mixed infections were detected in clinical sample testing.

**Table 7:** Test results of clinical samples.

Pathogens	Methods established in this study	Reference methods (Wongsawan et al., 2015; Xin et al., 2023)	Agreement
	Positive	Positive	
SS	5.77% (9/156)	5.77% (9/156)	100%
SS2	20.51% (32/156)	19.23% (30/156)	93.75%
SS7	8.33% (13/156)	5.77% (9/156)	69.23%
SS9	9.6% (15/156)	7.05% (11/156)	73.33%

## 4 Discussion

In this study, we developed a sensitive, specific and reproducible quadruplex TaqMan fluorescent quantitative PCR typing method based on the *gdh*, *cps2J*, *cps7J* and *cps9J* genes, which can differentiate between SS2, SS7, SS9 and other serotypes. The method does not cross-react with any of the non-target viruses or bacteria associated with infected pigs and accurately differentiates between SS2, SS7, SS9 and other serotypes of SS; The minimum detection limit for the recombinant plasmid standards P-SS and P-SS9 was 10 copies, and the minimum detection limit for P-SS2 and P-SS7 was 100 copies, which is comparable to the sensitivity of the quadruplex TaqMan fluorescence quantitative PCR assay for detecting SS and SS2 established by Xin L et al (Xin et al., 2023), whose method was only able to differentiate between SS2 and the other serotypes of SS. Coefficients of variation for both inter- and intra-batch replicates ranged from 0.21% to 1.10%. As there is no quadruplex TaqMan fluorescence quantitative PCR method that can differentiate between SS2, SS7, SS9 and other serotypes of SS, the establishment of this method is of great importance for rapid clinical typing and for guiding the precise use of medication and the timely adoption of appropriate measures.

Glutamate dehydrogenase (GDH), an important virulence-associated factor of SS, is expressed on the cell surface with NAD(P)H-dependent enzymatic activity and is a key enzyme linking carbon and nitrogen metabolism, which is important for bacterial pathogenicity (Chittick and Okwumabua, 2024). The study showed that the sequence of the *gdh* gene encoding glutamate dehydrogenase is highly conserved and that the gene is closely related to the reported *gdh* gene of SS (Silva et al., 2006). The nucleic acid sequences showed 97.2% to 98.4% homology with SS2, SS7 and SS9, of which 97.2% to 97.3% with SS2 strains, 98.4% with SS7 strains and 97.6% with SS9 strains (Smith et al., 1999; Okwumabua et al., 2001; Xu et al., 2021; Zhou et al., 2024). The amino acid sequences they encode were even more homologous, with more than 98.9% homology with sequences in GenBank (Okwumabua et al., 2001). Therefore, the *gdh* gene is a good target for the diagnosis of SS. CPS is the basis for distinguishing serotypes of SS (Goyette-Desjardins et al., 2020). Among them, *cps2J*, *cps7H* and *cps9J* are genes encoding specific podoplanet polysaccharide antigens of SS2, SS7 and SS9 respectively (Dekker et al., 2016; Jiang et al., 2022), which are closely related to the virulence and pathogenicity of SS and play an important role in bacterial survival and host infection. In addition, these genes are also used in molecular biology research for detection and analysis by PCR and other techniques to help understand the species of SS (Dekker et al., 2016; Wang et al., 2024), virulence factors and how they interact with the host, which is of great scientific importance and practical value in the prevention and control of porcine streptococcal infections.

The prevalence of *Streptococcus suis* in pigs is characterised by a wide spatial distribution that varies between regions and over time (Petrocchi Rilo et al., 2024; Uruén et al., 2024). According to the survey, 19 strains of SS were isolated from nine districts in Hubei Province in 2021-2023, including 10 serotypes, of which SS9 was the most prevalent, accounting for 21.05% of the total (Xia et al., 2024). Although SS2 was the most commonly reported strain infecting both humans and pigs, Wang M et al., showed that serotype 14 accounted for 71.1% and serotype 2 for 28.19% of ST-type SS isolates from Guangxi Zhuang Autonomous Region, China, during 2007-2018 (Wang et al., 2019). However, overseas, for example, testing of live pigs and pork at the Chiang Mai market in Thailand showed a positive rate of 84% for SS, with 34% positive for SS2, which remains the predominant serotype (Guntala et al., 2024). The most common serotypes of SS isolated from sick pigs in western Canada were SS2 (9.3%) and SS7 (7.8%) (de et al., 2024). Another study showed that the global prevalence of SS2 isolated from pigs was 13.6%, with approximately 10% in healthy pigs and 16% in sick pigs. The prevalence of SS2 did not change significantly over time. This suggests that SS2 continues to be a problem in the pig industry and a threat to human health (Keonam et al., 2024). In this study, the highest positivity rate was for SS2 (20.51%), followed bySS9 (9.6%) and SS7 (8.33%), while other serotypes of SS were also detected in the clinical samples tested (5.77%). In addition, the comparison with existing detection methods showed that the detection rate of the quadruplex TaqMan fluorescence quantitative PCR assay established in this study was higher than that of the conventional PCR method, and the concordance rate with the existing quadruplex TaqMan fluorescence quantitative PCR assay was higher. On the other hand, some of the samples were swabs, and although swabs are the easiest to collect, the nucleic acid content of the target pathogens was low after nucleic acid extraction, coupled with the limited sensitivity of conventional PCR, so there was a discrepancy in the detection rate of compliance between the two methods.

In conclusion, this study has established a sensitive, specific, rapid and efficient quadruplex TaqMan fluorescence quantitative PCR assay that can simultaneously differentiate between SS2, SS7, SS9 and other serotypes of SS, with the aim of providing technical support for rapid typing of SS and accurate prevention and treatment.

## Data Availability

The original contributions presented in the study are included in the article/supplementary material. Further inquiries can be directed to the corresponding authors.
